# Revaccination

**Published:** 1872-06

**Authors:** 


### REVACCINATION.

An infant should be vaccinated not earlier than a month after its birth; if it takes well, another careful vaccination about fourteen will be a life-long protection.

At a small-pox hospital in England, it is the invariable custom to revaccinate every nurse who enters the establishment to take care of the sick, and in thirty-four years there has not been a single case of a nurse having taken small-pox.

The older persons get after vaccination, the less likely are they to have any form of the disease.

As visitors to a place where contagious diseases are present are more liable to be infected than the regular inhabitants, it would seem to be a wise precaution of persons about taking a journey abroad to have a revaccination performed carefully, by the family physician, with reliable and fresh virus.

Those who are vaccinated with matter obtained directly from the cow, will certainly have a more complete exemption than those who use matter from the human subject; the protective power of the latter does wear out in time.

